# Changes in Cardiovascular Risk Factors and Health Care Expenditures Among Patients Prescribed Semaglutide

**DOI:** 10.1001/jamanetworkopen.2025.26013

**Published:** 2025-08-08

**Authors:** Yuan Lu, Yuntian Liu, Tedi Totojani, Chungsoo Kim, Rohan Khera, Hua Xu, John E. Brush, Harlan M. Krumholz, Jason Abaluck

**Affiliations:** 1 Section of Cardiovascular Medicine, Department of Internal Medicine, Yale School of Medicine, New Haven, Connecticut; 2 Center for Outcomes Research and Evaluation, Yale New Haven Hospital, New Haven, Connecticut; 3 Department of Biomedical Informatics and Data Sciences, Yale School of Medicine, New Haven, Connecticut; 4 Yale School of Management, New Haven, Connecticut; 5 Sentara Health Research Center, Sentara Health, Virginia Beach, Virginia; 6 Macon & Joan Brock Virginia Health Sciences at Old Dominion University, Norfolk, Virginia; 7 Department of Health Policy and Management, Yale School of Public Health, New Haven, Connecticut; 8 National Bureau of Economic Research, New Haven, Connecticut

## Abstract

**Importance:**

Semaglutide, a glucagon-like peptide-1 receptor agonist, has demonstrated substantial weight reduction and cardiovascular benefits in clinical trials. However, its association with clinical outcomes and health care expenditures remains underexplored.

**Objective:**

To evaluate changes in cardiovascular risk factors and health care expenditures among patients prescribed semaglutide across multicenter cohorts.

**Design, Setting, and Participants:**

This retrospective cohort study included 23 522 adults (≥18 years) who received an initial semaglutide prescription between January 1, 2018, and January 1, 2025, at Sentara Healthcare and between January 1, 2018, and May 1, 2025, at Yale New Haven Health System.

**Exposure:**

The first prescription of semaglutide identified at the ingredient level, stratified by type 2 diabetes status.

**Main Outcomes and Measures:**

Primary outcomes included changes in weight (% baseline), blood pressure (BP), total cholesterol, and hemoglobin A_1c_. Secondary outcomes assessed health care expenditures using Medicare-based cost estimates. A staggered difference-in-differences design compared outcomes of patients after the first semaglutide prescription with those of patients who had not yet received their prescription during the same period.

**Results:**

The study cohort included 23 522 patients (mean [SD] age at initiation of semaglutide, 56.2 [12.9] years; 66.7% female; 68.6% with diabetes). In the overall cohort, initiation of semaglutide was significantly associated with a −3.8% (95% CI, −3.9% to −3.7%) reduction in weight at 13 to 24 months, with a −5.1% (95% CI, −5.5% to −4.7%) reduction in weight among patients without diabetes. Significant reductions in diastolic BP (−1.5 mm Hg; 95% CI, −1.7 to −1.4 mm Hg), systolic BP (−1.1 mm Hg; 95% CI, −1.4 to −0.8 mm Hg), and total cholesterol (−12.8 mg/dL; 95% CI, −14.3 to −11.4 mg/dL) were also observed. Hemoglobin A_1c_ reductions were greater among patients with diabetes (−0.3%; 95% CI, −0.3% to −0.2%) compared with patients without diabetes (−0.1%; 95% CI, −0.2% to −0.01%). Imputed health care expenditures, excluding the cost of semaglutide, increased by $80 per month (95% CI, $68-$92 per month) during the 13- to 24-month period, which was associated with inpatient stays shifting to more expensive circulatory and metabolic diagnoses.

**Conclusions and Relevance:**

In this cohort study of adults prescribed semaglutide, initiation was associated with reductions in weight and cardiovascular risk factors but increases in health care expenditures, excluding semaglutide costs. These findings suggest potential clinical benefits in routine practice, while highlighting the need to evaluate the long-term impact of semaglutide on economic outcomes.

## Key Points

**Question:**

What are the changes in cardiovascular risk factors and health care expenditures associated with initiating semaglutide prescription?

**Findings:**

In this multicenter cohort study of 23 522 adults, semaglutide initiation was associated with significant reductions in weight, blood pressure, cholesterol, and hemoglobin A_1c_ levels at 13 to 24 months. However, semaglutide initiation was also associated with an increase in health care expenditures (not including the cost of semaglutide itself).

**Meaning:**

These findings suggest that, while semaglutide initiation is associated with improved cardiovascular risk profiles in clinical populations, its initiation is also associated with higher health care expenditures, underscoring the need to assess its long-term cost-effectiveness.

## Teaser

This cohort study evaluates changes in cardiovascular risk factors and health care expenditures after initiation of semaglutide.

## Introduction

Obesity, a complex neurometabolic disease, affects nearly half the US population and is significantly associated with morbidity and mortality.^1,2^ Recent advancements in pharmacotherapy for obesity, particularly antiobesity medications such as semaglutide, have demonstrated substantial weight reduction in clinical trials.^3,4^ As their use rapidly increases in clinical settings, these medications are being prescribed to diverse populations beyond those studied in trials and for extended periods.

Our goal in this study is to assess how clinical outcomes and health care expenditures change among patients prescribed semaglutide in clinical settings. Clinical trials, while valuable, involve controlled environments and selected populations that do not fully represent the broader, more diverse groups seen in routine clinical practice, where factors such as comorbidities, adherence behaviors, and socioeconomic status vary widely.^5,6^ Furthermore, it remains uncertain whether the broader use of semaglutide will increase or decrease health expenditures.

Accordingly, we assessed how cardiovascular risk factors and health care expenditures changed after semaglutide prescriptions using electronic health record (EHR) data from 2 large health systems: Sentara Healthcare System and Yale New Haven Health System (YNHHS). We compared changes in outcomes after the initial prescription of semaglutide with changes in outcomes during the same calendar months for patients who had not yet initiated semaglutide at that time but did so later. Our primary focus was weight change, with secondary analyses of blood pressure (BP), total cholesterol, hemoglobin A_1c_ (HbA_1c_), and health care expenditures. Our findings provide insights into the effectiveness and economic implications of semaglutide initiation in routine practice, enhancing the broader understanding of obesity management and its role in cardiovascular disease prevention.

## Methods

### Data Source

**Figure 1.**
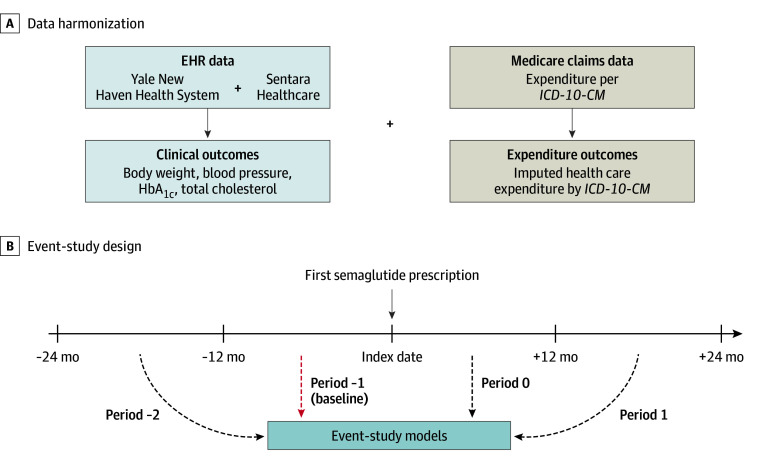
Study Overview Evaluation of how cardiovascular risk factors and health care expenditures changed after semaglutide prescriptions across multicenter cohorts in the Sentara Healthcare System and Yale New Haven Health System. EHR indicates electronic health record; HbA_1c_, hemoglobin A_1c_; and *ICD-10-CM*, *International Statistical Classification of Diseases and Related Health Problems, Tenth Revision, Clinical Modification*.

The EHR data sources consist of 2 large integrated health systems. Sentara Healthcare is a nonprofit health system with 12 hospitals serving Virginia and northeastern North Carolina, while YNHHS is an academic health system comprising 5 hospitals across Connecticut and Rhode Island. Both health systems serve a payer mix that includes patients with commercial insurance, Medicare, and Medicaid. Specific value-based or fee-for-service risk arrangements were not directly accounted for in this analysis. For both systems, key clinical data were extracted and transformed using the Observational Medical Outcomes Partnership common data model, version 5.3.19 (Figure 1). This retrospective cohort study was approved by the institutional review boards of Yale University and Sentara Healthcare, with a waiver for informed consent due to the use of deidentified retrospective data. This study followed the Strengthening the Reporting of Observational Studies in Epidemiology (STROBE) reporting guideline for cohort studies.

To complement the EHR data, Medicare claims data were incorporated to estimate health care expenditures. These claims provided cost estimates for *International Statistical Classification of Diseases and Related Health Problems, Tenth Revision, Clinical Modification* (*ICD-10-CM*) diagnostic codes, which were later merged with the diagnosis codes from the EHR. The Medicare data, available up to 2019, offered expenditure estimates for clinical encounters but excluded direct prescription costs, particularly for semaglutide, which was introduced in 2018. This integration enabled the assessment of health care use and expenditure trends associated with semaglutide prescriptions.

### Study Population

We identified adults (≥18 years) prescribed semaglutide between January 1, 2018, and January 1, 2025, in the Sentara system, and between January 1, 2018, and May 1, 2025, in YNHHS. Semaglutide initiation was defined as the first recorded prescription, regardless of formulation or dose, with the prescription date designated as the index date. To ensure sufficient data, patients were required to have at least 1 weight measurement in each of 4 periods: 13 to 24 months before the index date, 0 to 12 months before the index date, 0 to 12 months after the index date, and 13 to 24 months after the index date (Figure 1; eFigure 1 in Supplement 1). We tested the robustness of the findings using 2 alternative criteria: (1) requiring only 1 measurement in both the preindex and postindex periods and (2) requiring weight measurements every 6 months (eFigures 2 and 3 in Supplement 1). Patients with type 1 or type 2 diabetes were included; no exclusions were made based on prior use of glucagon-like peptide-1 receptor agonists. Analyses were stratified by type 2 diabetes status as a proxy for treatment intent—presuming weight management among those without diabetes and glycemic control among those with diabetes.

### Outcome Ascertainment

#### Clinical Outcomes

Clinical outcomes required at least 1 measurement per 12-month period, with mean values used per period. The primary outcome was the percentage weight change from baseline. Secondary outcomes included changes in BP, total cholesterol level, and HbA_1c_ level. The corresponding analysis cohort for each secondary clinical outcome represents a subset of the study population. Baseline values were calculated using mean values recorded during the 12 months preceding the index date.

#### Health Care Expenditure Outcomes

Health care expenditures were estimated by linking *ICD-10-CM* diagnostic codes from EHR data with Medicare-based cost estimates. Monthly expenditures were assigned based on the principal *ICD-10-CM* diagnosis using historical Medicare claims data, which reflect mean reimbursed amounts. These estimates were applied to all individuals, regardless of age or insurance type, and do not include billed charges or payer-specific variations (eMethods in Supplement 1).

For each unique (patient, month, primary diagnosis) combination in the Sentara or YNHHS data, we assigned expenditures based on matched Medicare-derived cost estimates, separately for inpatient and outpatient diagnoses. Nonsemaglutide drug costs were similarly imputed. Months without diagnoses were assigned zero expenditures. Baseline costs were calculated over the 12 months before the index date. This approach captured expenditure changes associated with visit frequency or diagnosis patterns but did not reflect cost variation within individual visits or diagnosis categories.

#### Medication Classification and Sociodemographic Variables

Semaglutide exposure was defined at the ingredient level, encompassing both injectable and oral formulations. In the absence of linked pharmacy fill data, we used an intent-to-treat approach based on prescription intent rather than confirmed use and did not disaggregate outcomes by formulation.

Sociodemographic variables, including age, sex, and race and ethnicity, were recorded at the index date. Data on sex and race and ethnicity were based on patient self-report using predefined categories from the Office of Management and Budget.^7^ These categories were included because they may be associated with both medication exposure and study outcomes.

### Statistical Analysis

We conducted event-study analyses to evaluate changes over time in key outcomes—weight, BP, total cholesterol level, HbA_1c_ level, and imputed monthly health care expenditures—after semaglutide initiation. In our baseline event-study models, data were divided into four 12-month periods relative to the prescription of semaglutide. Each outcome variable was regressed on indicators for these periods, including 2 periods prior to semaglutide prescription (a total of 2 years) and 2 periods after prescription (a total of 2 years) (Figure 1). The models accounted for calendar time fixed effects, individual random effects, and potential confounders, including age, sex, and race and ethnicity. Period effects were estimated separately for each database (Sentara Healthcare and YNHHS) and subsequently pooled using an inverse-variance weighting approach. We reported sensitivity analyses using alternative model specifications (eFigure 4 in Supplement 1). Specifically, we incorporated individual fixed effects, meaning that comparisons were made only within individuals over time, while restricting trends before semaglutide prescription to be zero.^8^ We found similar results in all cases.

Our design used a staggered adoption, 2-way fixed effects difference-in-differences approach rather than a traditional time-series model. By including calendar fixed effects, our model compared individuals who initiated semaglutide at different time points—for example, estimating the 12- to 24-month effect by comparing weight changes over the same calendar period between someone who started treatment in March 2017 and someone who started treatment in May 2019. Given the high rates of nonadherence and discontinuation reported in prior studies,^9,10,11^ we used EHR-based prescribing patterns to approximate persistence, including sensitivity analyses restricted to patients with 2 or more prescriptions or those with prescriptions 6 months or more apart. We also performed sensitivity analyses, adjusting for initiation or titration of antihypertensive and lipid-lowering medications, defined as new prescriptions or dosage changes within 6 months of semaglutide initiation, to assess potential confounding in cardiometabolic outcomes.

All *P* values were from 2-tailed tests, and results were deemed statistically significant at *P* < .05. Data extraction and preprocessing were conducted using Microsoft SQL Server (Microsoft Corp) and DuckDB (DuckDB Foundation), while statistical analyses were performed using R, version 4.2.1 (R Project for Statistical Computing).

## Results

### Patient Characteristics

This study included 23 522 patients (mean [SD] age at initiation of semaglutide, 56.2 [12.9] years; 66.7% female and 33.3% male; 13.4% Hispanic or Latino patients, 1.6% non-Hispanic Asian patients, 22.6% non-Hispanic Black patients, 59.5% non-Hispanic White patients, and 2.9% patients of other or unknown race and ethnicity) prescribed semaglutide who had at least 1 weight measurement in each of 4 distinct 12-month periods (16 813 patients from YNHHS; 6709 patients from Sentara Healthcare) between January 1, 2015, and the respective data end points—January 1, 2025, for Sentara and May 1, 2025, for YNHHS (eTable 1 and eFigure 1B in Supplement 1). The mean (SD) baseline body mass index (calculated as weight in kilograms divided by height in meters squared) was 37.7 (8.0). Of the cohort, 68.6% had diabetes as the presumed treatment indication.

Most patients had available measurements for BP (94.7%), HbA_1c_ level (53.0%), and total cholesterol level (35.7%). Most patients had multiple semaglutide prescriptions (mean [SD], 7.5 [6.3] prescriptions). A total of 82.7% had 2 or more semaglutide prescriptions, and 57.6% maintained active prescriptions at 13 to 24 months (eTable 1 in Supplement 1). Based on prescribing patterns, the estimated exposure durations were less than 3 months for 21.2% of patients, 3 to 6 months for 4.9%, 6 to 12 months for 6.8% of patients, and more than 12 months for 67.0% of patients.

### Association Between Semaglutide Prescription and Cardiovascular Risk Factors

**Figure 2.**
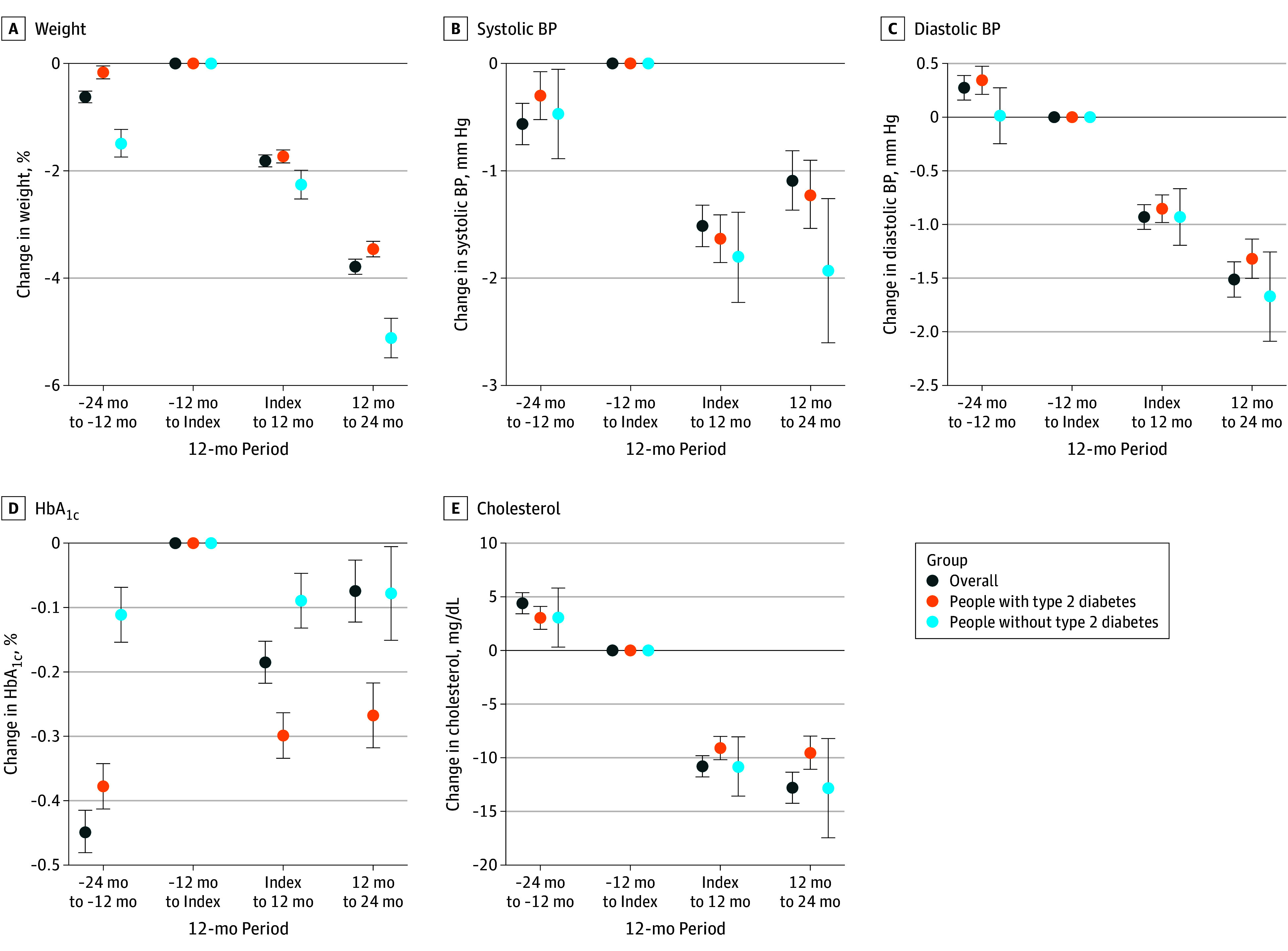
Changes From Baseline in Cardiovascular Risk Factors After Semaglutide Prescriptions, Overall and by Diabetes Status BP indicates blood pressure. Error bars indicate 95% CIs. SI conversion factors: To convert cholesterol to millimoles per liter, multiply by 0.0259; and HbA_1c_ to proportion of total hemoglobin, multiply by 0.01.

In the final 12-month follow-up period, semaglutide prescriptions were associated with significant improvements across multiple clinical parameters. The overall cohort experienced reductions in body weight (−3.8%; 95% CI, −3.9% to −3.7%), systolic BP (−1.1 mm Hg; 95% CI, −1.4 to −0.8 mm Hg), diastolic BP (−1.5 mm Hg; 95% CI, −1.7 to −1.4 mm Hg), total cholesterol level (−12.8 mg/dL; 95% CI, −14.3 to −11.4 mg/dL [to convert to millimoles per liter, multiply by 0.0259]), and HbA_1c_ level (−0.1%; 95% CI, −0.1% to −0.03% [to convert to proportion of total hemoglobin, multiply by 0.01]) (Figure 2, Table 1). Stratified analyses showed greater reductions in body weight and systolic BP among patients without diabetes than those with diabetes (body weight, −5.1% [95% CI, −5.5% to −4.7%] vs −3.5% [95% CI, −3.6% to −3.3%]; systolic BP, −1.9 mm Hg [95% CI, −2.6 to −1.3 mm Hg] vs −1.2 mm Hg; 95% CI, −1.5 to −0.9 mm Hg]). In contrast, patients with diabetes experienced greater reductions in HbA_1c_ level compared with those without diabetes (−0.3% [95% CI, −0.3% to −0.2%] vs −0.1% [95% CI, −0.2% to −0.01%]). The 24-month mean outcomes followed a similar pattern, with attenuated but directionally consistent associations reflecting the combination of early (0-12 months) and later (13-24 months) postprescription periods (Table 1).

**Table 1.** Changes in Body Weight and Cardiovascular Risk Factors After Semaglutide Prescriptions

Characteristic	Index body weight, % (95% CI)	BP, mm Hg (95% CI)		HbA_1c_, % (95% CI)	Total cholesterol, mg/dL (95% CI)
		Diastolic	Systolic		
**At 13-24 mo**					
Overall	−3.8 (−3.9 to −3.7)	−1.5 (−1.7 to −1.4)	−1.1 (−1.4 to −0.8)	−0.1 (−0.1 to −0.03)	−12.8 (−14.3 to −11.4)
With diabetes	−3.5 (−3.6 to −3.3)	−1.3 (−1.5 to −1.1)	−1.2 (−1.5 to −0.9)	−0.3 (−0.3 to −0.2)	−9.5 (−11.1 to −8.0)
Without diabetes	−5.1 (−5.5 to −4.7)	−1.7 (−2.1 to −1.3)	−1.9 (−2.6 to −1.3)	−0.1 (−0.2 to −0.01)	−12.9 (−17.5 to −8.2)
**At 1-24 mo**					
Overall	−2.1 (−2.2 to −2.0)	−0.9 (−1.1 to −0.8)	−1.5 (−1.7 to −1.3)	−0.2 (−0.2 to −0.2)	−10.6 (−11.5 to −9.6)
With diabetes	−2.1 (−2.2 to −2.0)	−0.9 (−1.0 to −0.8)	−1.6 (−1.8 to −1.4)	−0.3 (−0.3 to −0.3)	−9.0 (−10.1 to −8.0)
Without diabetes	−2.3 (−2.6 to −2.0)	−0.8 (−1.1 to −0.6)	−1.9 (−2.3 to −1.5)	−0.1 (−0.2 to −0.1)	−9.6 (−12.1 to −7.1)

Abbreviations: BP, blood pressure; HbA_1c_, hemoglobin A_1c_.

SI conversion factors: To convert total cholesterol to millimoles per liter, multiply by 0.0259; and HbA_1c_ to proportion of total hemoglobin, multiply by 0.01.

Sensitivity analyses assessed the association of varying measurement frequency, observation windows, modeling strategies, and data sources (eFigures 2, 4, and 5 in Supplement 1), demonstrating the robustness of results. Among the 74.2% of patients with continued therapy, slightly larger effect sizes were observed, consistent with a dose-response association (eFigures 7 and 8 in Supplement 1).

### Association Between Semaglutide Prescription and Health Care Expenditures

**Figure 3.**
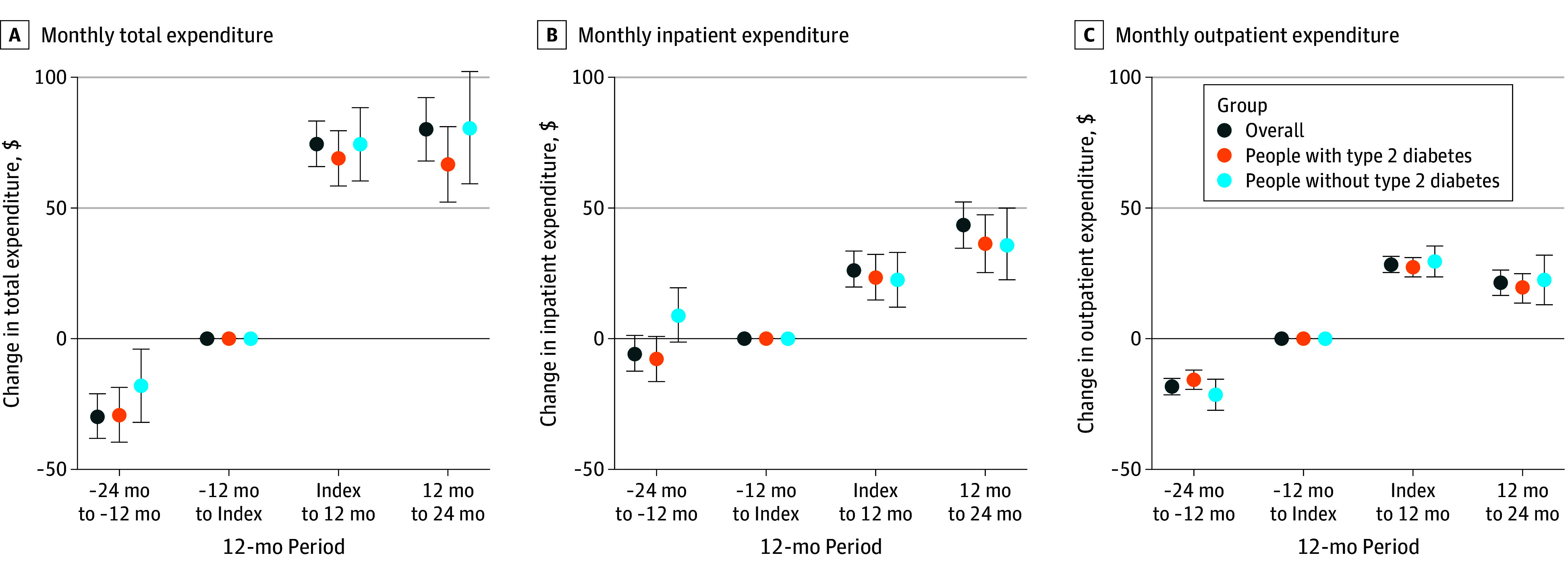
Changes From Baseline in Health Care Expenditure After Semaglutide Prescriptions, Overall and by Diabetes Status Error bars indicate 95% CIs.

Health care expenditures increased across multiple categories after semaglutide initiation. In the final 12-month period (months 13-24), total monthly expenditures increased by $80 (95% CI, $68-$92), with inpatient costs accounting for the largest share ($43; 95% CI, $34-$52) (Figure 3; Table 2). Increases were observed in both subgroups: $67 (95% CI, $52-$81) among patients with diabetes and $81 (95% CI, $59-$102) among those without diabetes (Figure 3; Table 2).

**Table 2.** Changes in Health Care Expenditure After Semaglutide Prescription^a^

Characteristic	Overall effect, $ (95% CI)			Breakdown by *ICD-10-CM* code, $ (95% CI)											
	Total	Inpatient	Outpatient	CD	D	E	F	G	H	I	J	K	L	M	N
**At 13-24 mo**															
Overall	80 (68 to 92)	43 (34 to 52)	21 (16 to 26)	5 (3 to 7)	1 (0 to 2)	21 (18 to 25)	0 (0 to 1)	1 (−1 to 2)	0 (0 to 0)	15 (10 to 19)	1 (−2 to 3)	6 (4 to 9)	2 (0 to 3)	2 (−1 to 5)	3 (1 to 5)
With diabetes	67 (52 to 81)	36 (25 to 47)	19 (13 to 25)	5 (2 to 8)	0 (−1 to 2)	11 (8 to 14)	0 (0 to 1)	1 (−1 to 3)	0 (0 to 1)	13 (8 to 19)	0 (−3 to 3)	6 (3 to 9)	1 (0 to 3)	1 (−3 to 5)	2 (0 to 4)
Without diabetes	81 (59 to 102)	36 (22 to 50)	22 (13 to 32)	3 (−1 to 8)	2 (0 to 4)	18 (13 to 23)	1 (−1 to 3)	2 (−2 to 5)	0 (−1 to 0)	11 (4 to 18)	−1 (−4 to 3)	8 (2 to 13)	0 (−2 to 2)	7 (1 to 13)	4 (1 to 7)
**At 1-24 mo**															
Overall	75 (66 to 83)	30 (24 to 37)	27 (23 to 30)	3 (1 to 5)	0 (−1 to 1)	21 (19 to 24)	0 (−1 to 1)	2 (0 to 3)	0 (0 to 1)	10 (6 to 13)	0 (−2 to 1)	5 (3 to 7)	1 (0 to 2)	4 (1 to 6)	4 (2 to 5)
With diabetes	70 (60 to 80)	27 (19 to 35)	26 (23 to 30)	3 (1 to 5)	0 (−1 to 1)	17 (15 to 20)	0 (−1 to 1)	1 (0 to 3)	0 (0 to 1)	9 (4 to 13)	−1 (−3 to 1)	5 (3 to 7)	0 (−1 to 2)	3 (0 to 6)	3 (2 to 5)
Without diabetes	72 (58 to 85)	23 (13 to 33)	26 (21 to 32)	3 (0 to 6)	1 (−1 to 2)	18 (14 to 21)	1 (0 to 2)	3 (0 to 5)	0 (−1 to 0)	8 (3 to 13)	−1 (−4 to 1)	6 (3 to 10)	0 (−1 to 2)	6 (2 to 11)	3 (1 to 5)

Abbreviations: CD, neoplasms; D, diseases of the blood and blood-forming organs and certain disorders involving the immune mechanism; E, endocrine, nutritional, and metabolic diseases); F, mental, behavioral, and neurodevelopmental disorders; G, diseases of the nervous system; H, diseases of the eye, adnexa, ear, and mastoid process; I, diseases of the circulatory system; *ICD-10-CM*, *International Statistical Classification of Diseases and Related Health Problems, Tenth Revision, Clinical Modification*; J, diseases of the respiratory system; K, diseases of the digestive system; L, diseases of the skin and subcutaneous tissue; M, diseases of the musculoskeletal system and connective tissue; N, diseases of the genitourinary system.

^a^ Estimated with primary diagnoses.

By diagnostic category, the largest expenditure increases were seen in diseases of the circulatory system ($15; 95% CI, $10-$19) and endocrine, nutritional, and metabolic diseases ($21; 95% CI, $18-$25) (Figure 3; Table 2). The magnitude of increases appeared to grow over time, although estimates were imprecise. Most diagnostic categories showed increasing expenditures, suggesting broad increases in health care use, especially for endocrine and cardiovascular conditions. As with clinical outcomes, sensitivity analyses for health care expenditures (eFigures 3 to 5 in Supplement 1) confirmed the consistency of findings.

## Discussion

In this multicenter cohort study, observed weight reductions in routine clinical practice were smaller than those reported in clinical trials (12-month change of –5.1% in our study vs –14.9% in the STEP [Semaglutide Treatment Effect in People with Obesity] 1 trial among patients with obesity but without diabetes).^3^ Patients with higher baseline weight and no diabetes diagnosis experienced the most pronounced benefits. Semaglutide initiation was also associated with improvements in BP, total cholesterol level, and HbA_1c_ level, underscoring its potential association with prevention of cardiovascular disease. However, we observed an initial increase in nonsemaglutide health care expenditures after semaglutide prescriptions, which, despite a slight decrease, remained elevated over the 24-month follow-up. These findings highlight a disconnect between clinical benefits and short-term cost savings, warranting caution when extrapolating trial-based projections to clinical settings.

Several factors may explain these findings. The reduction in cardiovascular risk factors after semaglutide prescriptions appears smaller than treatment effects estimated in clinical trials,^3,12,13^ which is consistent with broader differences between clinical populations and trial populations.^14,15,16^ However, methodological considerations were likely associated with conservative estimates, including reliance on EHR prescription records without pharmacy fill confirmation, pooling of oral and injectable formulations, and an intent-to-treat approach that did not account for discontinuation or nonadherence. The mean exposure duration of 6 to 7 months suggests many patients discontinued therapy earlier than in trials. In addition, increasing use of semaglutide via direct-to-consumer, cash-pay, or compounded sources—often outside EHR capture—along with supply constraints and payer variability, may have led to exposure misclassification. The relatively small proportion of patients without diabetes in our study (approximately 30%) likely reflects the midstudy approval of semaglutide for an indication of weight loss in 2021, as earlier semaglutide prescriptions were primarily indicated for type 2 diabetes.

The initial increase in health care expenditures may reflect increased care engagement after semaglutide initiation, including follow-up visits, monitoring, and management of adverse effects—potentially preceding any longer-term cost offsets from reduced hospitalizations. Our 24-month analysis did not demonstrate cost savings, in contrast to industry-sponsored models that project long-term savings (eg, over ≥10 years) based on trial-derived effectiveness.^17^ Additional factors may also be associated with increased expenditures. For instance, semaglutide may be initiated in response to emerging symptoms or worsening clinical status, which could be independently associated with health care use even in the absence of treatment. Such potential confounding underscores the need for further research to better isolate the causal effects of semaglutide on both clinical outcomes and health care costs in clinical settings.

Our study expands the prior literature in 3 important ways. First, it captures clinical outcomes across 2 health systems, providing insights into the generalizability of semaglutide’s associations beyond the controlled settings and selected populations of clinical trials. Second, our focus on cardiovascular risk factors, such as BP, cholesterol level, and HbA_1c_ level, extends the understanding of semaglutide’s potential for cardiovascular disease prevention, an area not fully explored in prior clinical studies. Finally, by leveraging both EHR and Medicare claims data, we obtained estimates of expenditure changes after semaglutide prescriptions. This comprehensive analysis highlights both the clinical benefits and the ongoing cost considerations, particularly as the use of antiobesity medications continues to increase.

Our findings have important public health and clinical implications. The clinical evidence of the association of glucagon-like peptide-1 receptor agonists with weight loss and cardiovascular risk reduction—particularly among patients without diabetes—suggests these agents may play a meaningful role in managing obesity, diabetes, and cardiometabolic risk more broadly.^18^ Clinically, this finding underscores the importance of integrating antiobesity medications into routine care, particularly for patients at higher risk of cardiovascular events. However, the increase in health care expenditures observed during the 24 months after semaglutide prescription underscores the challenge of balancing clinical benefits with short-term to medium-term financial sustainability in clinical settings.^19,20^ Our results call into question whether glucagon-like peptide-1 receptor agonists will “pay for themselves” through reduced expenditures of other kinds, at least in the short- to medium-term.

### Limitations

This study has several limitations. First, exposure was defined using EHR-recorded prescriptions without confirmation of pharmacy fills, which may have led to misclassification and biased estimates toward the null. However, most patients had multiple semaglutide prescriptions (mean [SD], 7.5 [6.3] prescriptions; 82.7% with ≥2 prescriptions), suggesting sustained use. Sensitivity analyses among persistent users showed larger effects, supporting robustness but highlighting differences from randomized clinical trials with ensured adherence. Second, we analyzed semaglutide at the ingredient level, combining oral and injectable forms to reflect clinical use and preserve power. This combination may have attenuated effect sizes given the lower effectiveness of oral formulations, although stratified analyses showed stronger effects with injectables. Third, our staggered difference-in-differences design leveraged variation in treatment timing to account for secular trends and time-invariant confounding. Accordingly, the validity of our estimates depends on the parallel trends assumption, and results should be interpreted with appropriate caution. If patients initiate semaglutide treatment earlier or more aggressively due to unobserved worsening of their underlying condition, our estimates could be biased upward—leading us to assume that increases in health care costs that may have occurred regardless of treatment were associated with semaglutide.

For expenditure analyses, we applied Medicare-allowed amounts, which likely underestimate true costs—particularly among commercially insured individuals. These estimates reflect diagnosis frequency and severity but not variation in reimbursement across payers or care models. Our data using a 24-month time window do not show cost savings. Although it is possible that such savings could be realized over a longer period, our study cannot confirm or rule out this possibility. We also did not account for variation in payment structures or risk contracts across health systems (eg, fee-for-service vs value-based care), which could be associated with patterns of health care use and generalizability of findings to other delivery systems. Although we adjusted for calendar time and individual effects, and sensitivity analyses accounting for changes in cardiometabolic medications yielded consistent results, residual confounding from unmeasured treatment changes remains possible. Finally, semaglutide obtained through direct-to-consumer platforms, cash-pay channels, or compounded sources outside the health systems may not have been captured, leading to potential underestimation of true exposure and uptake.

## Conclusions

In this cohort study of adults prescribed semaglutide, initiation was associated with significant improvements in weight, BP, cholesterol level, and glycemic control, particularly among those without diabetes, although the observed benefits were smaller than those reported in clinical trials. We observed a concomitant increase in health care expenditures over the 24 months after semaglutide prescriptions, underscoring the need for ongoing evaluation of its cost-effectiveness. Continued research is needed to determine semaglutide’s long-term impact on cardiovascular outcomes and whether broader use of this medication can achieve net reductions in health care expenditures over time.

## Notes

Supplement 1. **eMethods. **Imputation of Healthcare Expenditure in Electronic Health Record Data **eTable 1. **Baseline Characteristics of Pooled Patients Prescribed Semaglutide by Type 2 Diabetes **eTable 2. **Changes in Body Weight and Cardiovascular Risk Factors Following Semaglutide Prescription Across Alternative Specifications **eTable 3. **Changes in Healthcare Expenditure After Semaglutide Prescription Across Alternative Specifications **eFigure 1. **Flowchart Illustrating the Criteria to Define and Construct the Study Population **eFigure 2. **Sensitivity Analysis of Comparing 6-Month and 12-Month Specifications for Clinical Outcomes in the Overall Study Population **eFigure 3. **Sensitivity Analysis of Comparing 6-Month and 12-Month Specifications for Healthcare Expenditure Outcomes in the Overall Study Population **eFigure 4. **Sensitivity Analysis of Comparing Random Effects and Restricted Pre-Trends Models for Outcomes of Interest in the Overall Study Population **eFigure 5. **Sensitivity Analysis Comparing Different Data Sources (Sentara vs YNHHS) for Outcomes of Interest in the Overall Study Population **eFigure 6. **Monthly Inpatient and Outpatient Expenditure Over Age **eFigure 7. **Changes in Body Weight and Cardiovascular Risk Factors Following Semaglutide Prescription Across Alternative Specifications **eFigure 8. **Changes in Healthcare Expenditure After Semaglutide Prescription Across Alternative Specifications
eReference.

Supplement 2.
Data Sharing Statement
