# Supplementary material for: Changes in Cardiovascular Risk Factors and Health Care Expenditures Among Patients Prescribed Semaglutide
Source: JAMA Netw Open. 2025 Aug 8;8(8):e2526013. doi: 10.1001/jamanetworkopen.2025.26013 (PMC12334959; doi:10.1001/jamanetworkopen.2025.26013)
Supplement: Supplement 1. — eMethods. Imputation of Healthcare Expenditure in Electronic Health Record Data eTable 1. Baseline Characteristics of Pooled Patients Prescribed Semaglutide by Type 2 Diabetes eTable 2. Changes in Body Weight and Cardiovascular Risk Factors Following Semaglutide Prescription Across Alternative Specifications eTable 3. Changes in Healthcare Expenditure After Semaglutide Prescription Across Alternative Specifications eFigure 1. Flowchart Illustrating the Criteria to Define and Construct the Study Population eFigure 2. Sensitivity Analysis of Comparing 6-Month and 12-Month Specifications for Clinical Outcomes in the Overall Study Population eFigure 3. Sensitivity Analysis of Comparing 6-Month and 12-Month Specifications for Healthcare Expenditure Outcomes in the Overall Study Population eFigure 4. Sensitivity Analysis of Comparing Random Effects and Restricted Pre-Trends Models for Outcomes of Interest in the Overall Study Population eFigure 5. Sensitivity Analysis Comparing Different Data Sources (Sentara vs YNHHS) for Outcomes of Interest in the Overall Study Population eFigure 6. Monthly Inpatient and Outpatient Expenditure Over Age eFigure 7. Changes in Body Weight and Cardiovascular Risk Factors Following Semaglutide Prescription Across Alternative Specifications eFigure 8. Changes in Healthcare Expenditure After Semaglutide Prescription Across Alternative Specifications eReference. [file jamanetwopen-e2526013-s001.pdf]

Supplementary Online Content

Lu Y, Liu Y, Totojani T, et al. Changes in cardiovascular risk factors and health care expenditures among patients prescribed semaglutide. *JAMA Netw Open.* 2025;8(8):e2526013. doi:10.1001/jamanetworkopen.2025.26013

eMethods. Imputation of Healthcare Expenditure in Electronic Health Record Data
eTable 1. Baseline Characteristics of Pooled Patients Prescribed Semaglutide by Type 2 Diabetes
eTable 2. Changes in Body Weight and Cardiovascular Risk Factors Following Semaglutide Prescription Across Alternative Specifications
eTable 3. Changes in Healthcare Expenditure After Semaglutide Prescription Across Alternative Specifications
eFigure 1. Flowchart Illustrating the Criteria to Define and Construct the Study Population
eFigure 2. Sensitivity Analysis of Comparing 6-Month and 12-Month Specifications for Clinical Outcomes in the Overall Study Population
eFigure 3. Sensitivity Analysis of Comparing 6-Month and 12-Month Specifications for Healthcare Expenditure Outcomes in the Overall Study Population
eFigure 4. Sensitivity Analysis of Comparing Random Effects and Restricted Pre-Trends Models for Outcomes of Interest in the Overall Study Population
eFigure 5. Sensitivity Analysis Comparing Different Data Sources (Sentara vs YNHHS) for Outcomes of Interest in the Overall Study Population
eFigure 6. Monthly Inpatient and Outpatient Expenditure Over Age
eFigure 7. Changes in Body Weight and Cardiovascular Risk Factors Following Semaglutide Prescription Across Alternative Specifications
eFigure 8. Changes in Healthcare Expenditure After Semaglutide Prescription Across Alternative Specifications
eReference.

This supplementary material has been provided by the authors to give readers additional information about their work.

© 2025 Lu Y et al. *JAMA Network Open.*

eMethods. Imputation of Healthcare Expenditure in Electronic Health Record Data

To approximate costs using diagnoses, we make use of the Traditional Medicare Inpatient, Outpatient, Carrier, Skilled Nursing Facility, and Durable Medical Equipment files for the period 2016-2019. Inpatient expenditures are defined as expenses reported in the Inpatient, Skilled Nursing Facility, and Carrier claims with place of service code equal to 21, denoting an inpatient hospital facility. Outpatient expenditures are found in the Outpatient, Durable Medical Equipment, and Carrier claims with place of service code different from 21.

To get to the allowed amount associated with each claim, we add up the Medicare payment amounts along with any copays or deductibles. For the inpatient file, the Medicare payment amount is inclusive of any pass-through expenses. Diagnoses are operationalized using the ICD10 framework, and only principal diagnoses are considered. For the inpatient and outpatient estimates, we create a file with an observation for each beneficiary-month-diagnosis that we observe in any of the inpatient or outpatient files and then average expenditures across beneficiary-months. We also drop any expenditure amounts computed with eleven beneficiaries or less in compliance with data privacy rules. This process will understate expenditures related to diagnoses that are not present in the elderly (by virtue of using Medicare claims) and any expenditures related to COVID-19 (by virtue of limiting the estimation to the 2016-2019 period). This results in a match rate of 96% in Yale New Haven Hospital and 95% in Sentara Health.

To compute drug expenditures, we use total costs (not including any rebates or discounts from drug manufacturers) as observed in the Part D Event files spanning 2016-2019. Drug claims do not have associated diagnoses, so we use the ones in the inpatient or outpatient files—matching on the basis of beneficiary-month—and we subset to the most common ones for tractability. In other words, we create an observation for each beneficiary-month that we observe in the Part D Event file, we append the diagnoses dummies retrieved from the inpatient and outpatient files, and we regress the drug expenditures on the diagnosis dummies with beneficiary fixed effects. To reproduce these estimates on the Sentara end, we add up the betas associated with the diagnoses we observe for that month along with the constant.

While there is some concern regarding the incongruity of the age distributions between Medicare and Sentara, supplementary analysis (eFigure6) shows that expenditures associated with any given diagnosis decrease with age, contrary to what we might expect. We believe this is because older beneficiaries have a lower threshold for seeking medical care, so the medical issues associated with any given claim are less severe (and therefore less expensive). Additionally, commercial rates are often in excess of Medicare rates by as much as 241% (White & Whaley, 2021). Both of these factors likely lead us to underestimate expenditures as imputed in the Sentara data.

© 2025 Lu Y et al. *JAMA Network Open.*

eTable 1. Baseline Characteristics of Pooled Patients Prescribed Semaglutide by Type 2 Diabetes

**Characteristic**	**Overall**<br>N = 23,522	**People with type II diabetes**<br>N = 16,136	**People without type II diabetes**<br>N = 7,386
**Gender**			
Female	15,686 (66.7%)	9,713 (60.2%)	5,973 (80.9%)
Male	7,835 (33.3%)	6,422 (39.8%)	1,413 (19.1%)
Other/Unknown	1 (0.0%)	1 (0.0%)	0 (0.0%)
**Race & Ethnicity**			
Hispanic/Latino	3,163 (13.4%)	2,180 (13.5%)	983 (13.3%)
Non-Hispanic Asian	378 (1.6%)	295 (1.8%)	83 (1.1%)
Non-Hispanic Black	5,307 (22.6%)	3,836 (23.8%)	1,471 (19.9%)
Non-Hispanic White	13,991 (59.5%)	9,354 (58.0%)	4,637 (62.8%)
Other/Unknown	683 (2.9%)	471 (2.9%)	212 (2.9%)
**Age initiated Semaglutide**	56.2 (12.9)	58.7 (12.1)	50.8 (13.0)
**Index BMI**	37.7 (8.0)	37.3 (8.1)	38.5 (8.0)
**Index weight (kg)**	105.9 (25.2)	105.6 (25.3)	106.4 (25.0)
**Observed time span (years)**	9.4 (1.2)	9.5 (1.1)	9.3 (1.3)
**Total Semaglutide orders**	7.5 (6.3)	7.6 (6.2)	7.3 (6.6)
**Total Semaglutide orders by category**			
1	4,070 (17.3%)	2,607 (16.2%)	1,463 (19.8%)
2-6	8,300 (35.3%)	5,606 (34.7%)	2,694 (36.5%)
7-12	6,589 (28.0%)	4,809 (29.8%)	1,780 (24.1%)
>12	4,563 (19.4%)	3,114 (19.3%)	1,449 (19.6%)
**Length of exposure (months)**	24.2 (18.9)	26.7 (20.1)	18.7 (14.7)
**Length of exposure (months) by category**			
<3	4,995 (21.2%)	3,149 (19.5%)	1,846 (25.0%)
3-6	1,158 (4.9%)	712 (4.4%)	446 (6.0%)
7-12	1,602 (6.8%)	987 (6.1%)	615 (8.3%)
>12	15,767 (67.0%)	11,288 (70.0%)	4,479 (60.6%)
**First Semaglutide route**			
Oral	2,627 (11.2%)	2,182 (13.5%)	445 (6.0%)
Subcutaneous	20,895 (88.8%)	13,954 (86.5%)	6,941 (94.0%)
**Active Semaglutide orders in 13-24 months period**	13,553 (57.6%)	9,676 (60.0%)	3,877 (52.5%)
**Has concurrent anti-hypertensive meds**	13,563 (57.7%)	10,679 (66.2%)	2,884 (39.0%)
**Has concurrent anti-hyperlipidemic meds**	12,442 (52.9%)	10,461 (64.8%)	1,981 (26.8%)

n (%); Mean (SD),

© 2025 Lu Y et al. *JAMA Network Open.*

eTable 2. Changes in Body Weight and Cardiovascular Risk Factors Following Semaglutide Prescription Across Alternative Specifications

	% of index Weight	Diastolic BP (mmHg)	Systolic BP (mmHg)	HbA1c (%)	Cholesterol (mg/dL)
13-24m					
Main study results (N=23522)	-3.79 (-3.93, -3.65)	-1.51 (-1.68, -1.35)	-1.09 (-1.36, -0.81)	-0.07 (-0.12, -0.03)	-12.81 (-14.25, -11.37)
Two prescriptions (N=19444)	-3.96 (-4.12, -3.81)	-1.52 (-1.69, -1.34)	-1.21 (-1.5, -0.91)	-0.1 (-0.15, -0.05)	-13.23 (-14.76, -11.71)
Two prescriptions, 6 months apart (N=17451)	-4.01 (-4.17, -3.86)	-1.49 (-1.67, -1.3)	-1.27 (-1.58, -0.96)	-0.12 (-0.17, -0.07)	-13.57 (-15.15, -11.99)
Oral semaglutide only (N=2407)	-3.36 (-3.79, -2.93)	-0.8 (-1.44, -0.16)	-0.5 (-1.6, 0.6)	-0.11 (-0.29, 0.07)	-8.61 (-14.02, -3.2)
Subcutaneous semaglutide only (N=19021)	-3.91 (-4.06, -3.75)	-1.55 (-1.73, -1.37)	-1.18 (-1.47, -0.88)	-0.08 (-0.13, -0.03)	-13.01 (-14.54, -11.48)
Adjust for concurrent medications (N=23522)	-3.8 (-3.94, -3.66)	-1.56 (-1.72, -1.39)	-1.21 (-1.48, -0.94)	-0.09 (-0.13, -0.04)	-12.07 (-13.49, -10.64)
1-24m					
Main study results (N=23522)	-2.08 (-2.19, -1.98)	-0.94 (-1.06, -0.83)	-1.49 (-1.68, -1.3)	-0.21 (-0.24, -0.17)	-10.56 (-11.53, -9.6)
Two prescriptions (N=19444)	-2.14 (-2.25, -2.02)	-0.93 (-1.05, -0.8)	-1.51 (-1.72, -1.31)	-0.23 (-0.26, -0.19)	-10.97 (-11.99, -9.95)
Two prescriptions, 6 months apart (N=17451)	-2.21 (-2.32, -2.09)	-0.89 (-1.02, -0.76)	-1.51 (-1.72, -1.29)	-0.24 (-0.27, -0.2)	-11.41 (-12.47, -10.35)
Oral semaglutide only (N=2407)	-1.66 (-1.98, -1.35)	-0.6 (-0.99, -0.22)	-1.11 (-1.77, -0.44)	-0.17 (-0.27, -0.07)	-6.33 (-9.35, -3.31)
Subcutaneous semaglutide only (N=19021)	-2.09 (-2.21, -1.98)	-0.95 (-1.07, -0.82)	-1.46 (-1.67, -1.26)	-0.22 (-0.25, -0.18)	-11 (-12.05, -9.96)
Adjust for concurrent medications (N=23522)	-2.09 (-2.19, -1.98)	-0.97 (-1.08, -0.86)	-1.54 (-1.73, -1.35)	-0.21 (-0.24, -0.18)	-10.26 (-11.22, -9.3)

Values shown as: point estimate (95% CI).

© 2025 Lu Y et al. *JAMA Network Open.*

eTable 3. Changes in Healthcare Expenditure After Semaglutide Prescription Across Alternative Specifications

	Total expenditure ($)	Inpatient expenditure ($)	Outpatient expenditure ($)
13-24m			
Main study results (N=23522)	80 (68, 92)	43 (34, 52)	21 (16, 26)
Two prescriptions (N=19444)	72 (59, 84)	34 (25, 44)	19 (14, 24)
Two prescriptions, 6 months apart (N=17451)	67 (54, 80)	30 (21, 40)	18 (13, 23)
Oral semaglutide only (N=2407)	92 (57, 126)	35 (9, 62)	36 (22, 50)
Subcutaneous semaglutide only (N=19021)	71 (58, 85)	42 (32, 51)	13 (8, 19)
Adjust for concurrent medications (N=23522)	77 (65, 89)	42 (33, 50)	20 (15, 25)
1-24m			
Main study results (N=23522)	75 (66, 83)	30 (24, 37)	27 (23, 30)
Two prescriptions (N=19444)	67 (58, 76)	21 (14, 28)	24 (21, 27)
Two prescriptions6 months apart (N=17451)	62 (52, 71)	17 (10, 25)	23 (19, 26)
Oral semaglutide only (N=2407)	97 (73, 120)	40 (21, 59)	35 (26, 43)
Subcutaneous semaglutide only (N=19021)	64 (55, 74)	25 (18, 33)	21 (18, 25)
Adjust for concurrent medications (N=23522)	73 (64, 81)	29 (23, 36)	26 (23, 29)

Values shown as: point estimate (95% CI).

© 2025 Lu Y et al. *JAMA Network Open.*

eFigure 1. Flowchart Illustrating the Criteria to Define and Construct the Study Population

(A)

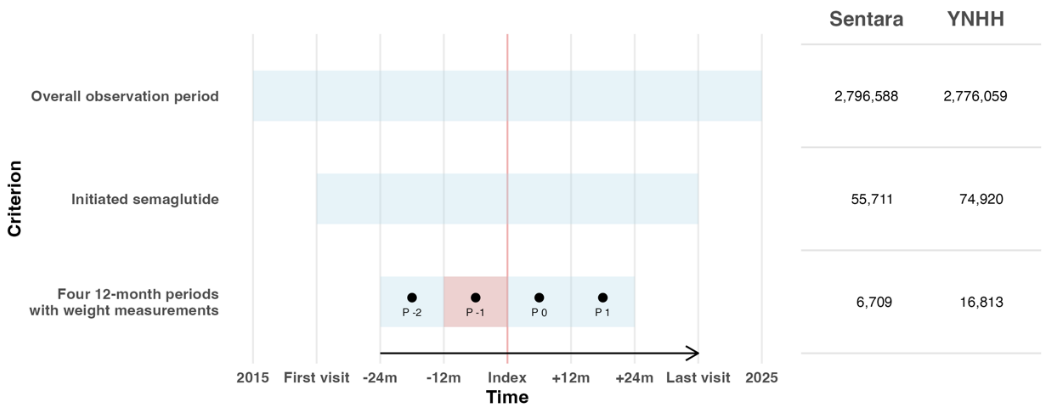

(B)

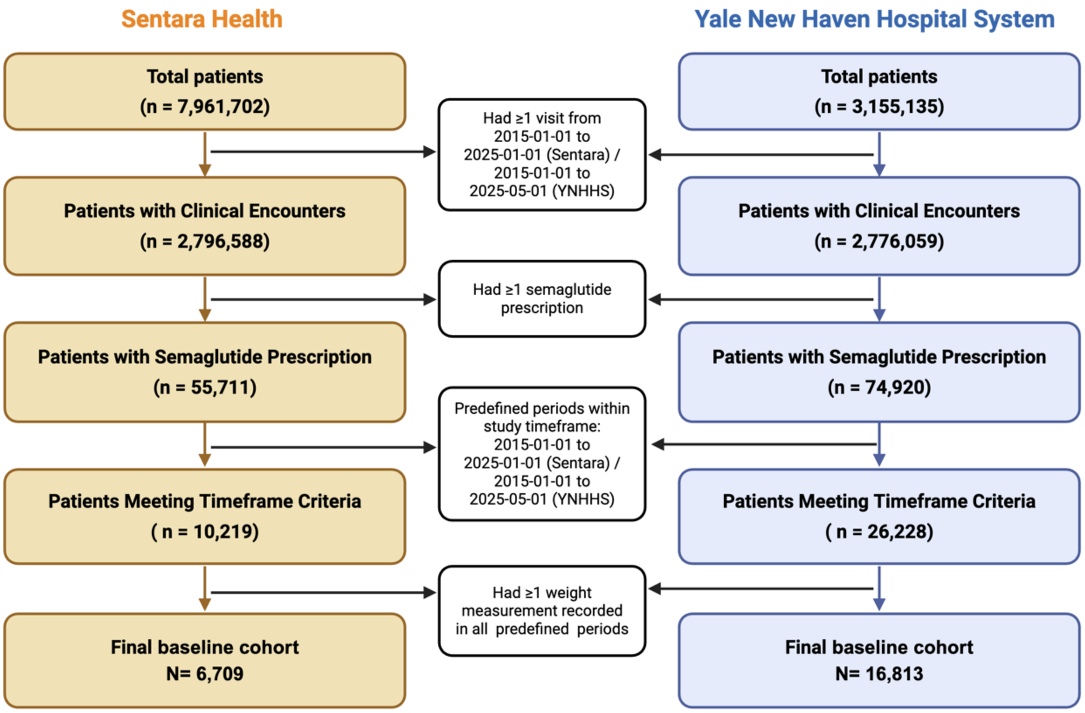

Footnote: Sentara: Sentara Healthcare System; YNHHS: Yale New Haven Health System.

© 2025 Lu Y et al. *JAMA Network Open.*

eFigure 2. Sensitivity Analysis of Comparing 6-Month and 12-Month Specifications for Clinical Outcomes in the Overall Study Population

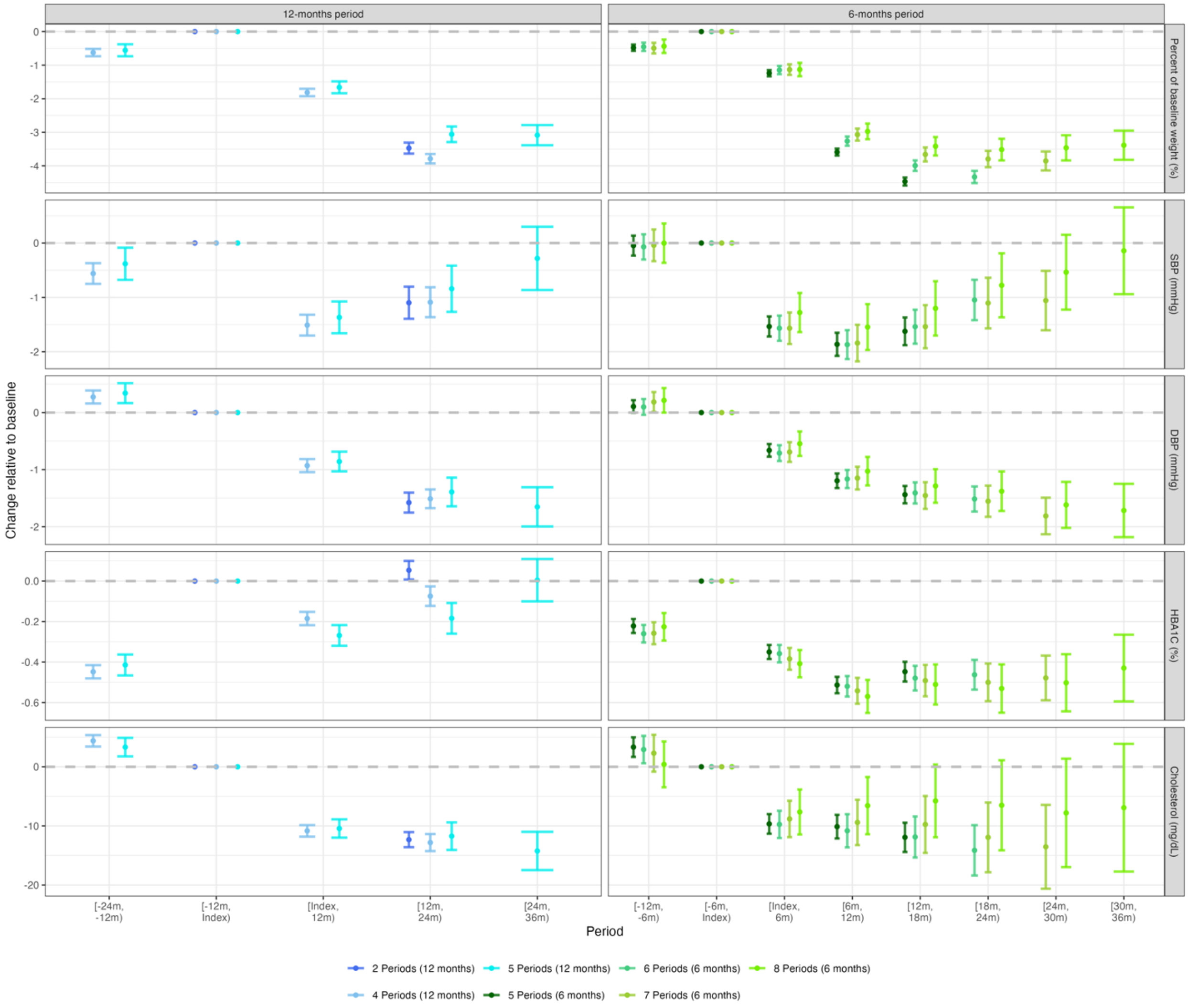

Footnote: Sensitivity analyses for clinical outcomes across varying period lengths and measurement frequencies. The left panels compare specifications requiring at least one clinical outcome measurement within different 12-month periods. Larger sample sizes were observed when fewer periods were required (For weight outcome: 4 periods, N = 23,522; 2 periods, N = 28,982; 5 periods, N = 10,924). The right panels assess stricter sampling (every 6 months) across varying follow-up lengths. Findings were consistent with the baseline, though precision declined in smaller, extended samples.

eFigure 3. Sensitivity Analysis of Comparing 6-Month and 12-Month Specifications for Healthcare Expenditure Outcomes in the Overall Study Population

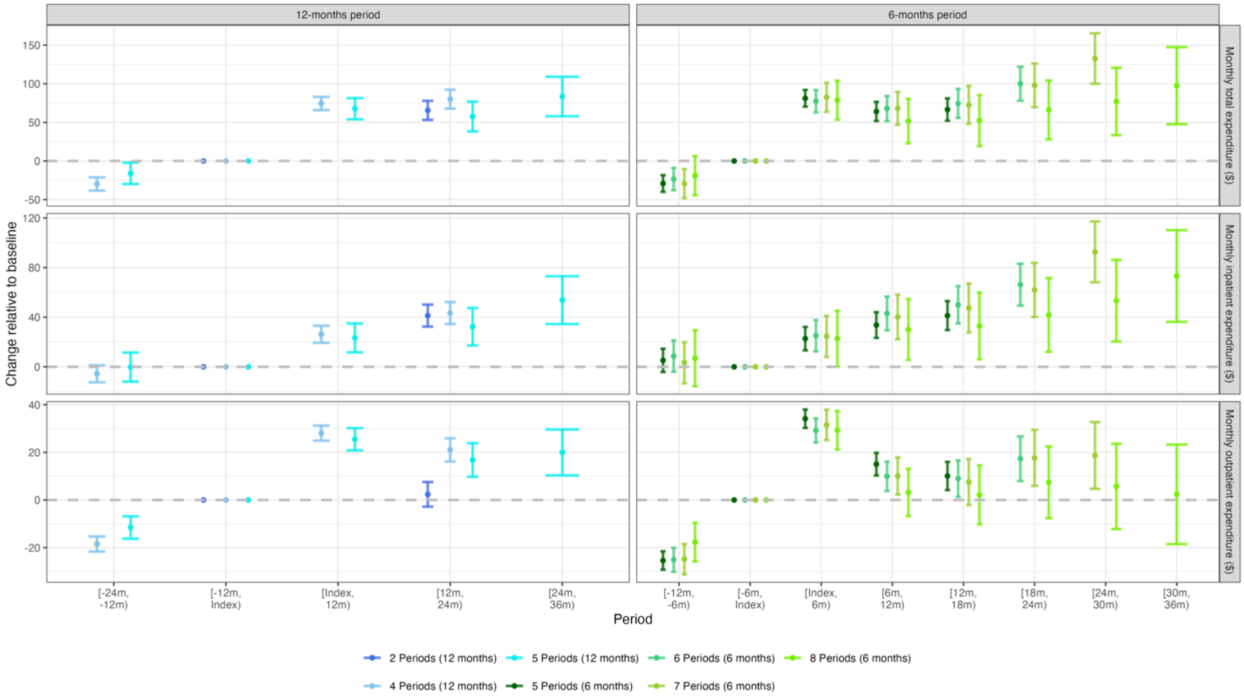

Footnote: Sensitivity analyses for expenditure outcomes across varying period lengths and measurement frequencies. The left panels compare specifications requiring at least one weight measurement within different 12-month periods for defining the study population. Larger sample sizes were observed when fewer periods were required (baseline: 4 periods, N = 23,522; 2 periods, N = 28,982; 5 periods, N = 10,924). The right panels assess stricter sampling (every 6 months) across varying follow-up lengths. Findings were consistent with the baseline, though precision declined in smaller, extended samples.

© 2025 Lu Y et al. *JAMA Network Open.*

eFigure 4. Sensitivity Analysis of Comparing Random Effects and Restricted Pre-Trends Models for Outcomes of Interest in the Overall Study Population

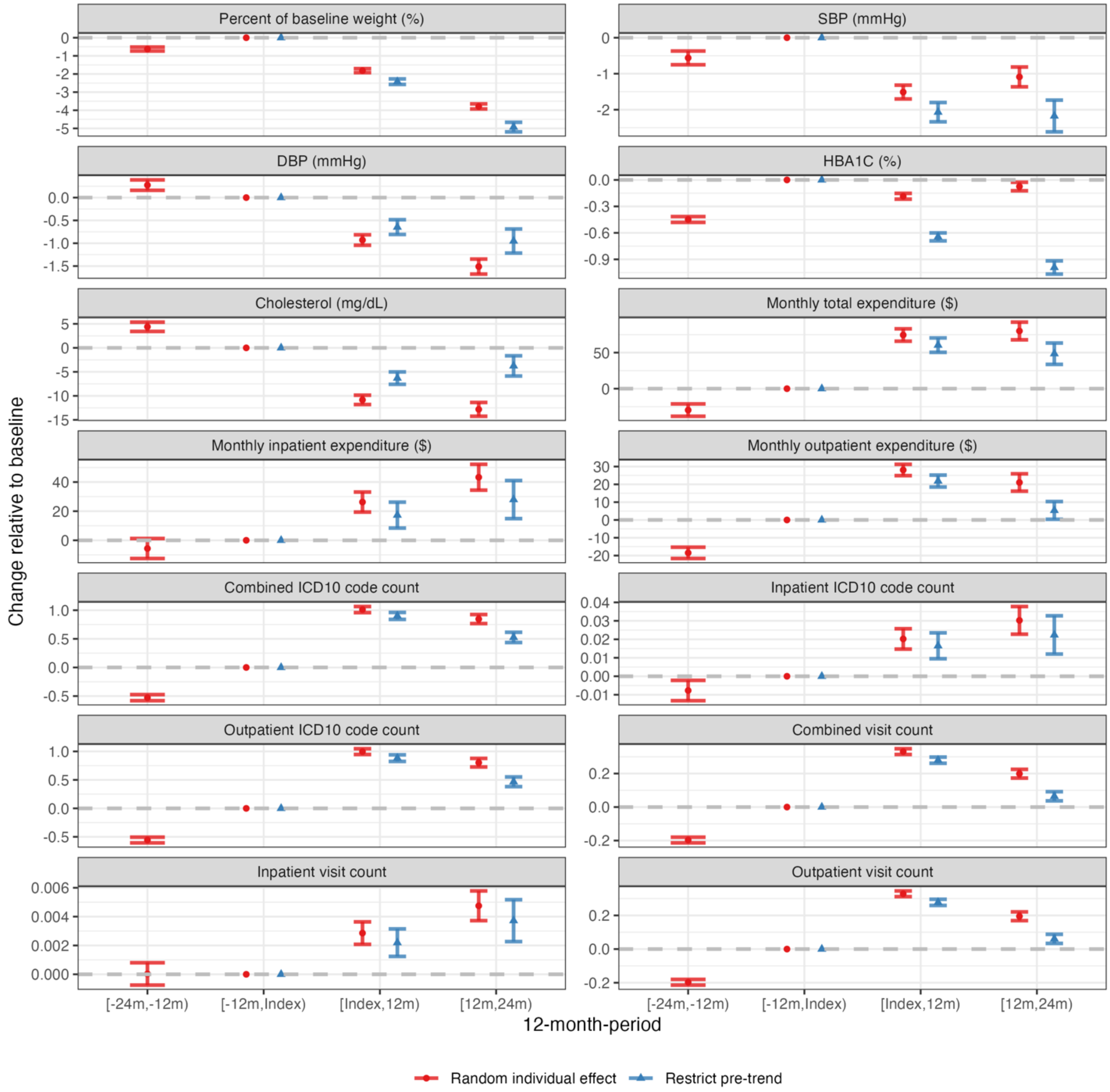

© 2025 Lu Y et al. *JAMA Network Open.*

eFigure 5. Sensitivity Analysis Comparing Different Data Sources (Sentara vs YNHHS) for Outcomes of Interest in the Overall Study Population

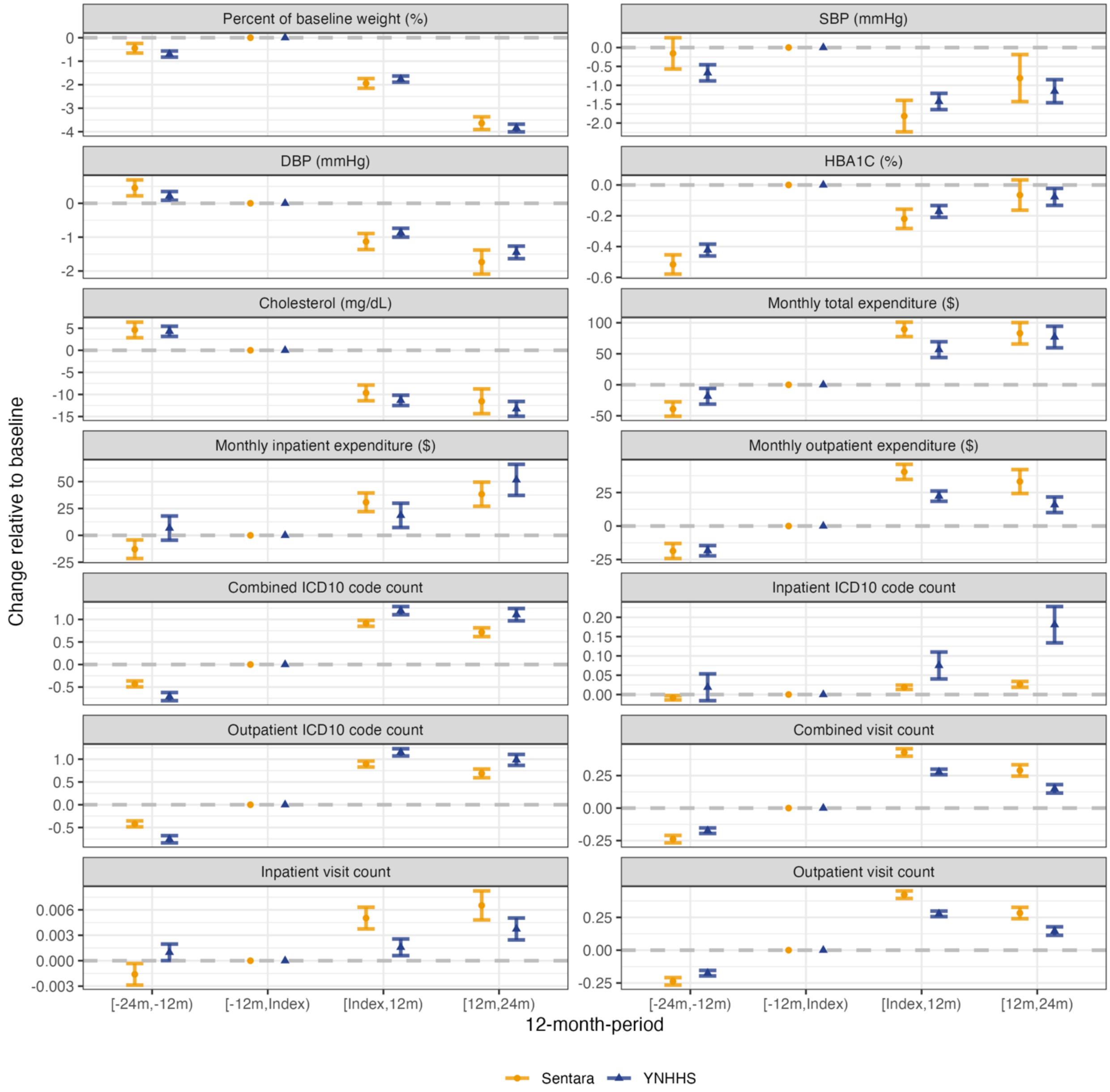

© 2025 Lu Y et al. *JAMA Network Open.*

eFigure 6. Monthly Inpatient and Outpatient Expenditure Over Age

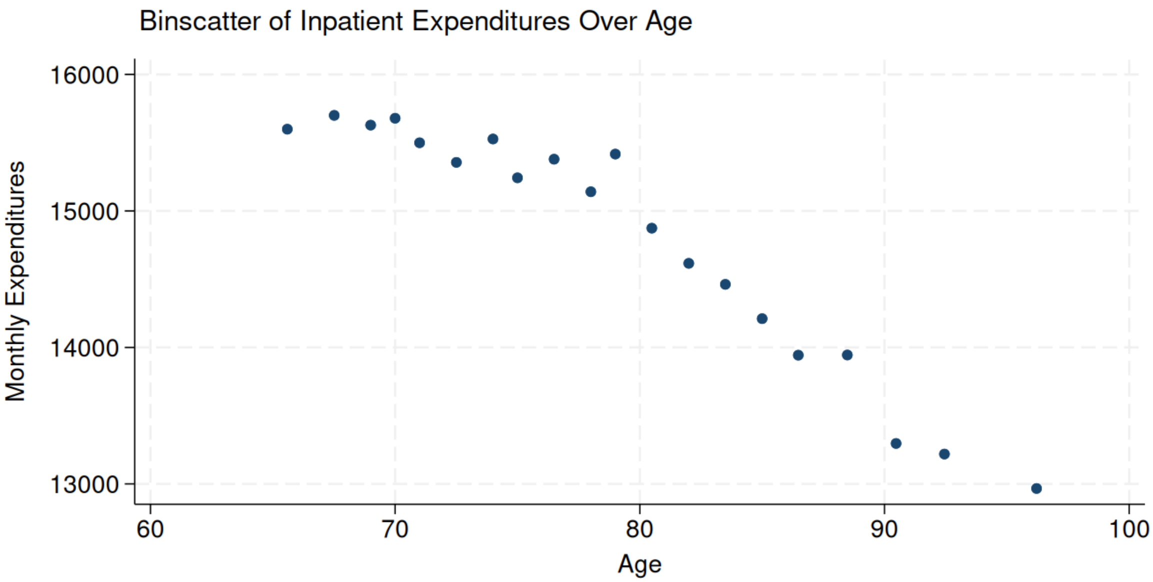

Notes:
a) Traditional Medicare inpatient files for 2016-2019.
b) Observations are at the level of beneficiary-month-year.
c) Conditional on observing at least one claim per beneficiary-month-year.

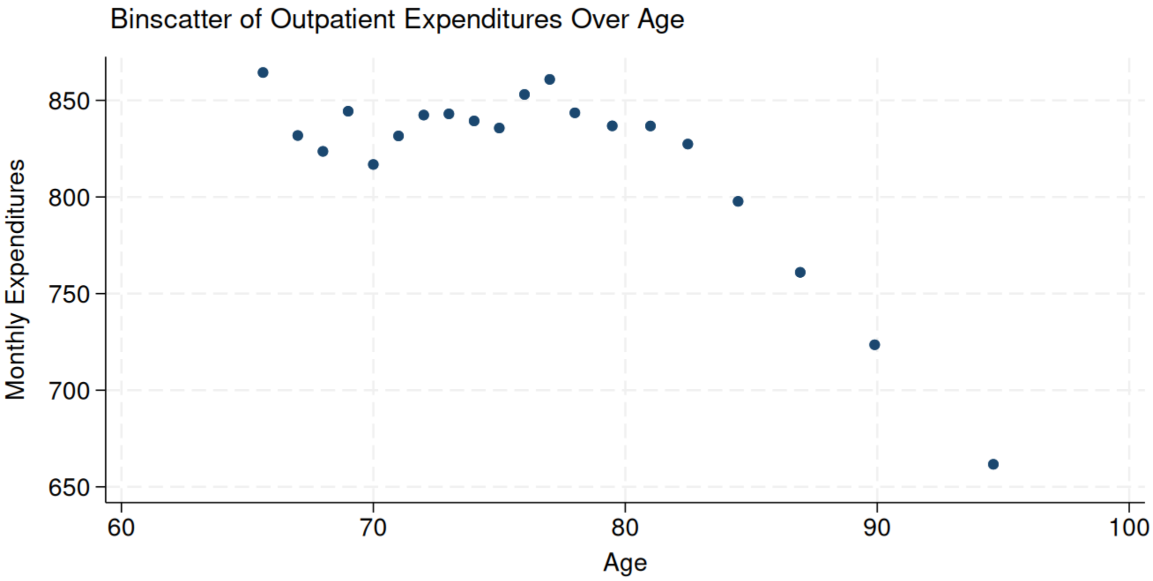

Notes:
a) Traditional Medicare outpatient files for 2016-2019.
b) Observations are at the level of beneficiary-month-year.
c) Conditional on observing at least one claim per beneficiary-month-year.

© 2025 Lu Y et al. *JAMA Network Open.*

eFigure 7. Changes in Body Weight and Cardiovascular Risk Factors Following Semaglutide Prescription Across Alternative Specifications

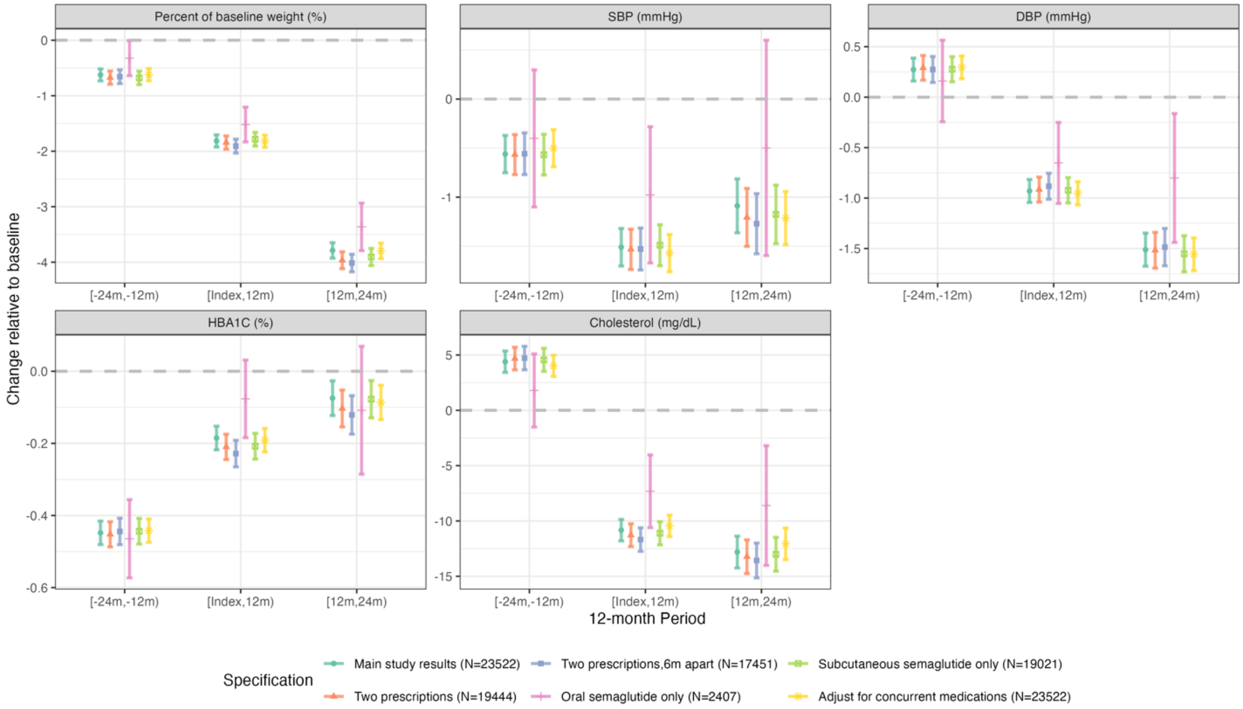

© 2025 Lu Y et al. *JAMA Network Open.*

eFigure 8. Changes in Healthcare Expenditure After Semaglutide Prescription Across Alternative Specifications

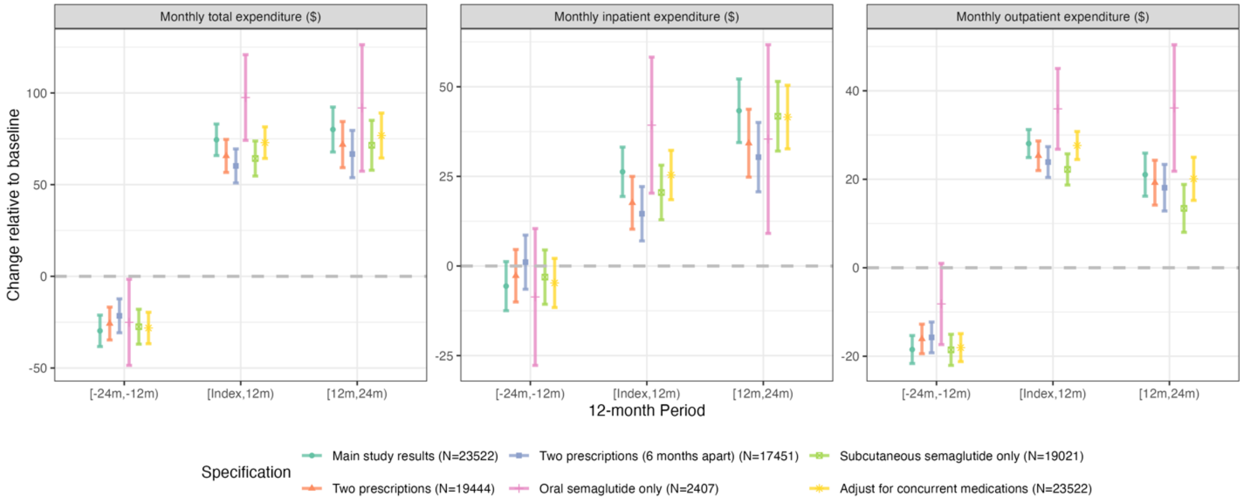

© 2025 Lu Y et al. *JAMA Network Open.*

eReference.

White, C., & Whaley, C. M. (2021). Prices paid to hospitals by private health plans are high relative to Medicare and vary widely: findings from an employer-led transparency initiative. *Rand Health Quarterly*, *9*(2), 5.

© 2025 Lu Y et al. *JAMA Network Open.*
