# Supplementary material for: Changes in Cardiovascular Risk Factors and Health Care Expenditures Among Patients Prescribed Semaglutide
Source: JAMA Netw Open. 2025 Aug 8;8(8):e2526013. doi: 10.1001/jamanetworkopen.2025.26013 (PMC12334959; doi:10.1001/jamanetworkopen.2025.26013)
Supplement: Supplement 2. — Data Sharing Statement [file jamanetwopen-e2526013-s002.pdf]

Data Sharing Statement

Lu. Changes in Cardiovascular Risk Factors and Health Care Expenditures Among Patients Prescribed Semaglutide. *JAMA Netw Open*. Published August 08, 2025. doi:10.1001/jamanetworkopen.2025.26013

Data
Data available: No

Additional Information
Explanation for why data not available: The data used in this study are derived from electronic health records at two large health systems and contain protected health information. Due to institutional and regulatory restrictions, we are unable to make individual-level patient data publicly available. Researchers interested in collaboration may contact the corresponding authors to discuss potential data use agreements or collaborative analyses.
